# Clustered Regularly Interspaced Short Palindromic Repeats/ CRISPR associated protein 9-mediated editing of *Schistosoma mansoni* genes: Identifying genes for immunologically potent drug and vaccine development

**DOI:** 10.1590/0037-8682-0131-2022

**Published:** 2022-08-12

**Authors:** Pragalathan Naidoo, Zilungile Lynette Mkhize-Kwitshana

**Affiliations:** 1 University of KwaZulu-Natal, College of Health Sciences, Department of Medical Microbiology, Durban, KwaZulu-Natal, South Africa.; 2 South African Medical Research Council (SAMRC), Division of Research Capacity Development, Cape Town, Western Cape, South Africa.

**Keywords:** CRISPR/Cas9-mediated gene editing, *Schistosoma mansoni* genes, Disease pathogenesis, Drug and vaccine development

## Abstract

Schistosomiasis is a neglected acute and chronic tropical disease caused by intestinal (*Schistosoma mansoni* and *Schistosoma japonicum*) and urogenital (*Schistosoma haematobium*) helminth parasites (blood flukes or digenetic trematodes). It afflicts over 250 million people worldwide, the majority of whom reside in impoverished tropical and subtropical regions in sub-Saharan Africa. Schistosomiasis is the second most common devastating parasitic disease in the world after malaria and causes over 200,000 deaths annually. Currently, there is no effective and approved vaccine available for human use, and treatment strongly relies on praziquantel drug therapy, which is ineffective in killing immature larval schistosomula stages and eggs already lodged in the tissues. The Clustered Regularly Interspaced Short Palindromic Repeats/CRISPR associated protein 9 (CRISPR/Cas9)-mediated gene editing tool is used to deactivate a gene of interest to scrutinize its role in health and disease, and to identify genes for vaccine and drug targeting. The present review aims to summarize the major findings from the current literature reporting the usage of CRISPR/Cas9-mediated gene editing to inactivate genes in *S. mansoni* (*acetylcholinesterase (AChE)*, *T2 ribonuclease omega-1 (ω1)*, *sulfotransferase oxamniquine resistance protein (SULT-OR)*, and *α-N-acetylgalactosaminidase (SmNAGAL)*), and freshwater gastropod snails, *Biomphalaria glabrata* (*allograft inflammatory factor (BgAIF)*), an obligatory component of the life cycle of *S. mansoni*, to identify their roles in the pathogenesis of schistosomiasis, and to highlight the importance of such studies in identifying and developing drugs and vaccines with high therapeutic efficacy.

## INTRODUCTION

Neglected tropical diseases (NTDs) affect over 2 billion people worldwide and thrive in tropical and subtropical climate regions in Asia, Africa, and the Americas, which are heavily burdened by poverty, lack of clean water supplies, poor sanitation, inadequate healthcare facilities, and infectious disease vectors^1^. According to the World Health Organization (WHO), schistosomiasis (bilharzia), soil-transmitted helminthiasis, Buruli ulcer, cholera, Chagas disease (American trypanosomiasis), dracunculiasis (guinea-worm disease), dengue fever, leprosy (Hansen's disease), human African trypanosomiasis (sleeping sickness), leishmaniasis, onchocerciasis (river blindness), lymphatic filariasis (elephantiasis), and trachoma are currently the major NTDs affecting the global population^2^. The clinical and epidemiological features and currently available treatment regimens for the aforementioned NTDs are summarized in Table 1.

**TABLE 1:** Clinical and epidemiological features of the major neglected tropical diseases afflicting the global population in 2021 according to the WHO.

Disease/ infection	Causative agent	Prevalence	(i) Treatment regimen/(ii) Ailments	References
Schistosomiasis (Bilharzia) (helminth infection)	- Various *Schistosoma* worms: - Intestinal (*S. mansoni, S. japonicum)* - Urogenital (*S. haematobium*)	- Affects over 250 million people globally - 779 million people are at risk globally - Sub-Saharan Africa, southeast Asia, the middle East, and the Caribbean	(i) Praziquantel, Oxamniquine (ii) Katayama fever, lymphocytosis, central nervous system disorders, genital sores, and organ fibrosis	^5^
Soil-transmitted helminthiasis: - Ascariasis - Trichuriasis - Strongyloidiasis - Ancylostomiasis - Necatoriasis (helminth infections)	*- Ascaris lumbricoides (AL)* *- Trichuris trichiura (TT)* *- Strongyloides stercoralis (SS)* *- Ancylostoma duodenale (AD)* - *Necator americanus (NA)*	- Affect 1.5 billion people globally - *AL*: 1.2 billion - *TT*: 795 million - *SS*: 600 million - *AD* + *NA*: 740 million - Sub-Saharan Africa, China, the Americas, and East Asia	(i) Albendazole, Mebendazole (ii) Anemia, cutaneous lesions, and respiratory and gastrointestinal tract infections	^21^
Buruli ulcer (bacterial infection)	*- Mycobacterium ulcerans*	- Around 2270-5000 cases/ year globally - West Africa and Australia	(i) Combination of Rifampicin and Clarithromycin/ or Moxifloxacin, Telacebec (ii) Nodules and necrotizing lesions	^22^
Cholera (bacterial infection)	*- Vibrio cholerae*	- 1.3-4 million cases/year globally - 21,000-143,000 deaths/year globally - Africa, the Caribbean, and South/ Southeast Asia	(i) Doxycycline, Azithromycin, Ciprofloxacin (ii) Nausea, vomiting, diarrhea, dehydration, and muscle cramps	^23^
Chagas disease (American trypanosomiasis) (protozoan infection)	*- Trypanosoma cruzi*	- Affect 6-8 million people globally - 50,000 deaths/ year globally - 65-100 million people are at risk globally - Mostly the Americas and some areas in Africa, Eastern Mediterranean, and Western Pacific	(i) Benznidazole, Nifurtimox (ii) Cardiomyopathy, gastrointestinal tract infection, and central nervous system disorder	^24^
Dracunculiasis (Guinea-worm disease) (helminth infection)	*- Dracunculus medinensis*	- Around 54 cases/year globally - Africa	(i) No specific drug available; Aspirin or Ibuprofen used to reduce pain and inflammation (ii) Pruritic and painful blisters	^25^
Dengue fever (viral infection)	- Arbovirus (Flavivirus family)-infected mosquitoes (*Aedes aegypti and Aedes albopictus*)	- Affects over 100 million people globally - Over 40,000 deaths/ year globally - Africa, Eastern Mediterranean, the America, South-East Asia, and Western Pacific	(i) No specific drug available; Acetaminophen to control pain and fever; Aspirin, Ibuprofen, and Naproxen sodium not recommended (ii) Myalgia, petechial rash, fever, and gastrointestinal tract bleeding	^26^
Leprosy (Hansen's disease) (bacterial infection)	*- Mycobacterium leprae*	- Over 200,000 cases/year globally - Over 2 million people permanently disabled - Africa, Asia, and the Americas	(i) Combination of Rifampicin, Dapsone, Clofazimine (ii) Cutaneous lesions, and damage to the nerves, skin, limbs, and eyes	^27^
Human African trypanosomiasis (Sleeping sickness) (protozoan infection)	*- Trypanosoma brucei* (*T. b. gambiense* and *T. b. rhodesiense* subsp.)-infected tsetse fly (*Glossina* spp.)	- Over 1,000 cases/year globally - Africa	(i) Pentamidine, Suramin, Melarsoprol, Eflornithine, Fexinidazole (ii) Lethargy, central nervous system disorders, and lymphadenitis	^28^
)Leishmaniasis (protozoan infection)	*- Leishmania* spp.-infected female sandflies (*Phlebotomus* and *Lutzomyia* spp.)	- Affects 700,000-1,000,000 people globally - 20,000-30,000 deaths/year globally - Africa, the Americas, and South East Asia	(i) Liposomal amphotericin B, Miltefosine, Paromomycin, Pentamindine (ii) Ulcerative cutaneous lesions	^29^
Onchocerciasis (River blindness) (helminth infection)	*- Onchocerca volvulus*-infected blackflies (*Simulium* spp.)	- Affects over 20.9 million people globally - Skin disease: 14.6 million, vision loss: 1.15 million - Sub-Saharan Africa, and some areas in the Americas and Middle East	(i) Ivermectin, Moxidectin (ii) Blindness, cutaneous pigmentation, and atrophy	^30^
Lymphatic filariasis (Elephantiasis) (helminth infection)	*- Wuchereria bancrofti* and *Brugia malayi*-infected mosquitoes	- Affects over 51 million people globally - 859 million people are at risk globally - Africa, Asia, the Western Pacific, and the Caribbean	(i) Diethylcarbamazine (ii) Lymphedema, and connective tissue and skin edema	^31^
Trachoma (bacterial infection)	*- Chlamydia trachomatis*	- Affects over 40 million people globally - 137 million people are at risk globally - Over 1.9 million have blindness or visual impairments - Africa, Asia, the Americas, Australia, and the Middle East	(i) Azithromycin (ii) Blindness, eye inflammation, eyelid scarring, trichiasis, and corneal clouding	^32^

Schistosomiasis is an acute and chronic disease caused by intestinal (*Schistosoma mansoni* and *Schistosoma japonicum*) and urogenital (*Schistosoma haematobium*) helminth parasites, often referred to as blood flukes (digenetic trematodes)^3^. Adult worms reside in the mesenteric veins of the abdomen (*S. mansoni* and *S. japonicum*) and the venous plexus of the urinary bladder (*S. haematobium*)^3^. As an obligatory component of the life-cycle of *S. mansoni*, sporocysts adhere to freshwater gastropod snails to promote larval development^4^.

Approximately 250 million people worldwide, 90% of whom live in sub-Saharan Africa, are infected with schistosomes through contact with larval forms (cercariae) present in freshwater, and over 779 million people are at the risk of infection^5^. In terms of prevalence and socio-economic impacts of NTDs, schistosomiasis ranks second in the list of most common parasitic diseases, with malaria being at the top of the list, with an annual mortality rate of over 200,000^3^.

The pathogenesis of schistosomiasis is associated with the manifestation of cercarial dermatitis, genital sores, Katayama fever (characterized by urticarial rash, fever, enlarged spleen and liver, and bronchospasm), lymphocytosis, central nervous system disorders, periportal fibrosis, and ultimately death^3^. Currently, no approved vaccine is available to prevent schistosomiasis, and treatment relies heavily on praziquantel drug therapy, which is ineffective in killing immature intra-mammalian larval schistosomula stages and eggs already dislodged in the tissues, does not prevent reinfection, and parasite resistance to praziquantel continues to remain an ever-growing threat^6^ ^-^ ^10^. In addition, the cure rate of praziquantel therapy ranges between 60% and 90%^11^. For the successful elimination of schistosomiasis, innovative medical and technological advancements along with strengthened multi-pronged interventions such as (i) raising awareness, (ii) continual surveillance, (iii) early infection detection, (iv) mass deworming programs, (v) proper sanitation and clean water provision, (vi) allocation of more research funds, (vii) development of sensitive diagnostic tools, (viii) new therapeutic agents, and (ix) prophylactic vaccines, are needed^12^.

The Clustered Regularly Interspaced Short Palindromic Repeats/CRISPR-associated protein 9 (CRISPR/Cas9)-mediated gene editing tool plays an invaluable role in identifying genes involved in resistance to toxins, drugs, or infectious diseases as well as in interrogating the functionality of genes in health and diseases^13^. This method of gene editing requires two crucial components, namely CRISPR-associated protein 9 (Cas9), a DNA-cutting protein from *Streptococcus pyogenes* that localizes and binds to the protospacer adjacent motif (PAM) sequence in the genome, and a single guide RNA (sgRNA) consisting of CRISPR RNA (crRNA; approximately 17-20 nucleotides long nucleotide sequence that is complementary to the target DNA) and trans-activating CRISPR RNA (tracrRNA; a binding scaffold for Cas9). Both Cas9 and sgRNA bind together to form a ribonucleoprotein complex capable of identifying and cutting specific sections of DNA in the gene of interest^14^ ^,^ ^15^. The DNA repair machinery (non-homologous end joining (NHEJ), homology directed repair (HDR), and DNA polymerase theta-mediated end joining (TMEJ)) tends to be error-prone, leading to mutations that can disable the overall functionality of the gene^14^ ^,^ ^15^.

**FIGURE 1:**
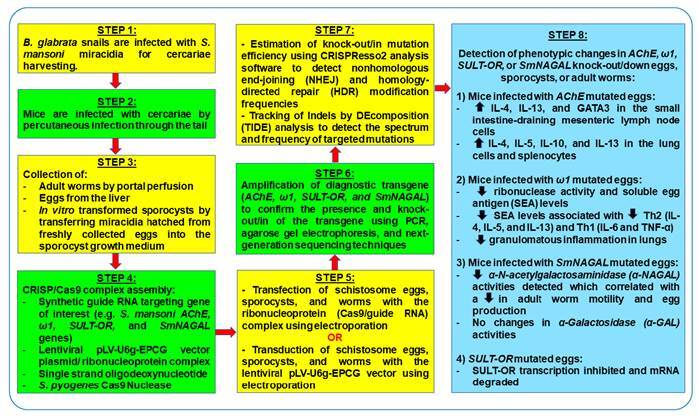
Overview of the key steps involved in executing the CRISPR/Cas9 gene editing technique to inactivate *AChE*, *ω1*, *SULT-OR*, and *SmNAGAL* genes in *S. mansoni*, and the subsequent phenotypic changes^16^ ^-^ ^20^.

Recently, CRISPR/Cas9-mediated gene editing tools have proven to be useful in understanding the pathophysiological, metabolic, and immunological mechanisms underlying the manifestation and severity of schistosomiasis as well as in highlighting the importance of developing vaccines and drugs with high therapeutic efficacy (Table 2) (Figure 1)^4^ ^,^ ^16^ ^-^ ^20^. This review aims to summarize the major findings from the current literature reporting the usage of the CRISPR/Cas9-mediated gene editing tool to inactivate genes in *S. mansoni* to identify the functional role of these genes in disease pathogenesis and to highlight the importance of developing vaccines and drugs with high therapeutic efficacy to target disease-associated genes.

**TABLE 2:** Summary of studies that used the CRISPR/Cas9 gene editing technique to inactivate various genes in *S. mansoni*.

Gene	Aims/Objectives	Major findings	Ref
*Acetylcholinesterase (AChE)* gene: - Chromosome 1. - Tegumental outer membrane and in musculature. - Functional roles in neuromuscular cholinergic system, regulation of glucose and nutrient scavenging, sexual maturation, and reproduction.	- To investigate the use of CRISPR/Cas9-mediated genome editing to mutate *AChE* gene (by targeting two gRNAs, X5 and X7, located on exons 5 and 7 of *Smp_154600*, respectively) in *S. mansoni* eggs, and its influence on soluble egg antigen (SEA) levels and Th1/ Th2 cytokine production.	- Modification frequency: majority (HDR) and rare (NHEJ). - Mutated eggs displayed diminished SEA levels and 8.3%-10.7% reduction in *AChE* activity. - Mice infected with *AChE*X5/KI-edited mutated eggs: enhanced Th2 immune responses in the small intestine-draining mesenteric lymph node cells (evident by the elevated expression of IL-4, IL-13, and GATA3), and lung cells and splenocytes (evident by the elevated expression of IL-4, IL-5, IL-10, and IL-13). - Mice infected with *AChE*X7/KI-edited mutated eggs: no significant changes.	^16^
*Omega-1 (ω1)* gene: - Chromosome 1. - Glycosylated T2 ribonuclease. - Hepatotoxic tissue destroying egg antigen. - Functional roles in Th2 polarization and granuloma formation. - Enhances inflammasome-dependent IL-1β activation and secretion in macrophages.	- To investigate the use of CRISPR/Cas9-mediated genome editing to mutate the *ω1* gene (by targeting gRNA located on exon 6 *Smp_193860*) in *S. mansoni* eggs, and its influence on soluble egg antigen (SEA) levels and Th1/Th2 cytokine production.	- Modification frequency: NHEJ (~4.5%) and HDR (0.19%). More than 98% of NHEJ modified reads were substitutions. - Mutated eggs displayed diminished ribonuclease activity and SEA levels. - Diminished SEA levels associated with reduced Th2 (IL-4, IL-5, and IL-13) and Th1 (IL-6 and TNF-α) cytokine levels. - No changes in IL-2 and IL-10 levels. - IFN-γ was not detected in wildtype and mutated eggs. - Mice infected with mutated eggs displayed reduced granulomatous inflammation in lungs.	^18^
	- To compare the efficiency of RNA-guided AsCas12a nuclease from *Acidaminococcus* sp. with SpCas9 from *Streptococcus pyogenes* to improve the mutagenesis and transgene knock-in efficiency of CRISPR in AT-rich genomes of *S. mansoni* eggs (by targeting ω1 copies located at nucleotide positions 3,980,364 to 3,984,675 (*Smp_334170.1, Smp_334170.2*), 3,992,964 to 3,995,246 (*Smp_334240*), and 3,908,953 to 3,911,250 (*Smp_3339*30)).	- Cleavages catalyzed by SpCas9 and AsCas12a resulted in blunt- and staggered-ended strand breaks, respectively. - Tracking of Indels by DEcomposition (TIDE) analysis showed that AsCas12a was more efficient than SpCas9 for gene KO. - CRISPResso2 analysis showed that most mutations were deletions. - AsCas12a groups had the greatest KO efficiency with NHEJ % of: (i) SpCas9 plus ssODN (15.67%), (ii) AsCas12a plus T-ssODN (21.43%), (iii) AsCas12a plus NT-ssODN (28.71%). - SpCas9 group had the greatest transgene insertion/ KI efficiency: (i) KI_AsCas12a-T-ssODN (12.37%), (ii) KI_AsCas12a NT-ssODN (14.58%), and (iii) KI_SpCas9 (17.07%).	^20^
*Sulfotransferase oxamniquine resistance protein (SULT-OR)* gene: - Chromosome 6. - Membrane-bound SULTs: are found in the Golgi apparatus and affect functional properties of lipids, proteins, peptides, and glycosaminoglycans. - Cytosolic SULTs: involved in the defense system through metabolizing endogenous substrates. - Oxamniquine: antischistosomal prodrug that binds to *S. mansoni SULT* known as *smSULT-O*R.	- To investigate the CRISPR/Cas9 efficiency during the parasitic stages of *S. mansoni* (eggs, mother sporocysts (the first intramolluscan stage), and adult worms) by introducing somatic mutations in the *SULT-OR* gene (located on exon 1 of *Smp_089320*) with the aim of developing an oxamniquine-resistant stable KO transgenic cell line to test and identify oxamniquine derivates for antischistosomal activity.	- Next-generation sequencing: adult worms had 10 times more reads containing deletion mutations (0.3-2.0% of aligned reads) compared to sporocysts (0.1-0.2%). - Deletion mutations were extremely rare in eggs. - Most common deletion in adults and sporocysts was a 34 bp deletion directly upstream of the predicted cut site. - Homozygous (biallelic) deletions caused oxamniquine resistance by causing frameshifts, inhibiting *SULT-OR* transcription, or leading to mRNA degradation via the nonsense-mediated mRNA decay pathway. - *SULT-OR* knock-down was not observed at the mRNA level.	^17^
*α-N-acetylgalactosaminidase (SmNAGAL)* gene: - Lysosomal exoglycosidase enzyme. - Crucial for α-galactosidase/α-NAGAL substrate cleavage and activity. - Role in maintaining cellular homeostasis by regulating glycan substrates on carbohydrates, lipids, and proteins.	- To investigate whether *S. mansoni* contains functionally important glycosyl hydrolase family 27 orthologs of *Homo sapiens α-Galactosidase (α-GAL)* and *α-N-acetylgalactosaminidase (α-NAGAL*) proteins.	- Five glycosyl hydrolase family 27 members were found in *S. mansoni.* - Only one, termed *SmNAGAL* (*Smp_089290*), contained all 13 ligand binding amino acid residues and both catalytic aspartic acid residues that are necessary for *α-GAL/α-NAGAL* substrate cleavage and activity. - Both *α-GAL* and *α-NAGAL* enzymatic activities were higher in females compared to those in males (*α-NAGAL* > *α-GAL*). - *α-GAL* and *α-NAGAL* were abundant in neuronal and parenchymal cells as well as in the vitellaria and mature vitellocytes of the adult schistosome.	^19^
	- To investigate the functional role of the newly discovered *SmNAGAL* gene (*Smp_089290*) in *S. mansoni* adult worms by using small interfering RNA (siRNA)-mediated knockdown and programmed CRISPR/Cas9 somatic genome editing to inhibit its activity.	- siRNA-mediated knockdown (> 90%) in adult worms significantly inhibited *α-NAGAL* activity. - Detectable levels of genome editing: 0.25%-0.31%. - No significant reduction in *α-GAL* activity. - Decrease in *α-NAGAL* activity correlated with inhibition of adult worm motility and egg production. - *Smp_089290* acted predominantly as an *α-NAGAL* (*SmNAGAL*) and is thought to play a role in coordinating movement and oviposition processes.	^19^

**α-GAL:** alpha-galactosidase (α-GAL); **α-NAGAL:** alpha-N-acetylgalactosaminidase; **ω1:** T2 ribonuclease omega-1; **AChE:** acetylcholinesterase; **AsCas12a:** nuclease from *Acidaminococcus* sp.; **AT-rich:** adenine and thymine-rich; **GATA3:** GATA binding protein 3; **gRNA:** guide RNA; HDR: homology directed repair; **IFN-γ:** interferon gamma; **IL:** interleukin; **KI:** knock-in; **KO:** knockout; **NHEJ:** nonhomologous end joining; **NT-ssODN:** non-CRISPR target-single-stranded oligodeoxynucleotide; **SEA:** soluble egg antigen; **siRNA:** small interfering RNA; **smNAGAL:** *S. mansoni* α-N-acetylgalactosaminidase; **SpCas9:** nuclease from *Streptococcus pyogenes*; **SULT-OR:** sulfotransferase oxamniquine resistance protein; **Th1:** T-helper type 1; **Th2:** T-helper type 2; **TNF-α:** tumor necrosis factor alpha; **T-ssODN:** target-single-stranded oligodeoxynucleotide.

## CRISPR/CAS9-MEDIATED EDITING OF *S. MANSONI* GENES FOR DRUG AND VACCINE DEVELOPMENT

### *S. mansoni* acetylcholinesterase (AChE) gene

In humans, acetylcholinesterase (AChE), a cholinergic enzyme, is predominantly found at the postsynaptic neuromuscular junctions; it hydrolyzes acetylcholine, a naturally occurring neurotransmitter found in the central and peripheral nervous systems, into choline and acetic acid, leading to the termination of neurotransmission at cholinergic synapses^33^. In adult schistosomes, AChE is found on the tegumental outer membrane and in the musculature^34^ ^-^ ^36^; it plays a vital role in the neuromuscular cholinergic system^37^ ^,^ ^38^, regulation of glucose scavenging from the host blood^39^, control of muscle functionality^35^, and activities such as feeding and scavenging of nutrients from the host, sexual maturation, and reproduction^35^. AChE suppression indirectly affects female fecundity, leading to the release of immature eggs in increased numbers and reduced sizes of liver granulomas. In male worms, AChE plays a crucial role in regulating metabolic functions^38^. Schistosome eggs and host cells involved in advanced granuloma formation around parasite eggs entrapped in organs and tissues also contain AChE. The secretion of schistosome egg AChE contained within the eggs was reported to inhibit the host IL-4 immune response and, as a result, played a role in granuloma formation, which was further advanced by the host cell AChE^38^. Several approved and marketed anthelmintics that directly target AChE with varying degree of effectiveness, are currently available^40^ ^-^ ^42^. AChE is a desired target for future drug and vaccine development with high therapeutic efficacy since there is no cross-reactivity between schistosome and human AChE^35^, and it acts through targeting parasite muscle ion-channels (peptide gated chloride Cl^ˉ^ channels, Ca^2+^activated Cl^ˉ^ channels, Ca^2+^ channels, and K^+^ channels), which open when acetylcholine interacts with the acetylcholine receptor (nAChR)^40^.

You *et al*. (2021) used the gene knock-out and knock-in (KI) method to deactivate the 56.78 kb *AChE* gene (*Smp_154600*) located on chromosome 1 in *S. mansoni* eggs^16^. In mice infected with AChEX5/KI-edited schistosome eggs, T-helper type 2 (Th2) immune responses were significantly enhanced in lung cells and splenocytes (evident by the elevated expression of interleukin (IL)-4, IL-5, IL-10, and IL-13), and in small intestine-draining mesenteric lymph node cells (evident by the elevated expression of IL-4, IL-13, and GATA3) when compared to control mice infected with unmutated eggs. No other significant changes were noted in mice infected with AChEX7/KI-edited schistosome eggs^16^.

### *S. mansoni* T2 ribonuclease omega-1 (ω1) gene

Omega-1 (ω1), a glycosylated T2 ribonuclease, is a major tissue-destroying and Th2-inducing soluble egg antigen contained in or secreted by *S. mansoni* eggs, and it is important for Th2 polarization^43^ ^-^ ^46^ and granuloma formation^18^. ω1 is hepatotoxic^47^, and is responsible for conditioning dendritic cells for Th2 polarization by limiting the interaction of dendritic cells with CD4^+^ T lymphocytes^43^. Everts *et al.* (2012) found that both ω1 ribonuclease activity and glycosylation as well as the ability of ω1 to suppress protein synthesis following internalization by the mannose receptor, condition dendritic cells for Th2 priming^44^. ω1 also enhances inflammasome-dependent IL-1β secretion in macrophages stimulated with Toll-like receptor 2 ligand and has the ability to regulate several pattern-recognition receptor signaling pathways^45^. Lewis X (LeX), also known as stage-specific embryonic antigen-1 or CD15, which is a glycan motif located on ω1, contributes to the Th2-inducing properties of ω1^46^. Granulomatous inflammation around the eggs passing through the intestinal wall and those trapped in the host organs and hepatic sinusoids is stimulated by ω1, promoting fibrosis that ultimately results in hepatointestinal schistosomiasis^18^ ^,^ ^48^ ^,^ ^49^.

Ittiprasert *et al*. (2019) deactivated the 6,196 bp-long T2 ribonuclease *ω1* gene (*Smp_193860*) found on chromosome 1 in *S. mansoni* eggs^18^. The eggs with mutated ω1 displayed diminished ribonuclease activity and soluble egg antigen (SEA) levels compared to the unmutated eggs. Diminished SEA levels were associated with significantly reduced Th2 (IL-4, IL-5, and IL-13) and Th1 (IL-6 and TNF-α) cytokine levels, whereas no significant changes in IL-2 and IL-10 levels were noted. IFN-γ was not detected in either the wild-type or the mutated eggs. In mice injected with the mutated eggs, granulomatous inflammation was noticeably reduced in the lungs compared with that observed in the control mice infected with the unmutated egg^18^.

Ittiprasert *et al*. (2022) compared the efficiency of RNA-guided AsCas12a nuclease from *Acidaminococcus* sp. with that of SpCas9 from *Streptococcus pyogenes* to improve the mutagenesis and transgene knock-in efficiency of CRISPR in schistosomes with AT-rich sequence of the *ω1* gene^20^. Both enzymes preferred to mutate the reference *Smp_334170* copy as well as *Smp_334240* over *Smp_333930* and/or *Smp_333870 ω1* copies located on chromosome 1 in *S. mansoni* eggs. Programmed cleavages catalyzed by Cas9 and Cas12a resulted in blunt- and staggered-ended strand breaks, respectively. Compared to SpCas9, AsCas12a demonstrated superior programmed knockout activity at a specific target site in the *ω1* gene, as determined by Tracking of Indels by DEcomposition (TIDE) analysis. CRISPResso2 analysis showed that most mutations were deletions. Both nucleases performed similarly well in terms of HDR-based transgene insertion. Although AsCas12a caused fewer mutations per genome than SpCas9, the phenotypic impacts of both nucleases on *ω1* transcription and expression were similar. It was concluded that AsCas12a could be used more frequently in functional AT-rich genomes such as those found in schistosomes^20^.

### *S. mansoni* sulfotransferase oxamniquine resistance protein (SULT-OR) gene

In humans, sulfotransferases (SULTs) catalyze the transfer of a sulfuryl group (SO_3_) from the ubiquitous sulfate donor, 3'-phosphoadenosine-5'-phosphosulphate (PAPS), to an acceptor amine (R-NH_2_) or alcohol (R-OH) substrate to form sulfamate (R-NH-SO_3_^-^) or sulfate (R-O-SO_3_^-^) products, respectively^50^. Membrane-bound SULTs found in the Golgi apparatus and sulfonates affect the structural and functional characteristics of lipids, proteins, peptides, and glycosaminoglycans^51^ ^,^ ^52^. Cytosolic SULTs are involved in defense mechanisms by metabolizing small endogenous substrates (bile acids, steroids, and neurotransmitters). On the other hand, they also metabolically activate xenobiotics, such as N-hydroxy heterocyclic amines, N-hydroxy arylamines, and hydroxymethyl polycyclic aromatic hydrocarbons, leading to the production of highly reactive electrophiles that are both carcinogenic and mutagenic^51^ ^,^ ^52^.

Prodrugs are activated and metabolized by sulfated substrates in the body into pharmacologically active compounds with pathological consequences, including cell death^50^. Oxamniquine is an antischistosomal prodrug activated within schistosome parasites, and it is a species-specific treatment effective against only *S. mansoni* adult worms^50^ ^,^ ^53^. Oxamniquine induces its killing effect by binding to a specific *S. mansoni* SULT, known as smSULT-OR, where it is transiently sulfated. Once activated, oxamniquine binds to DNA and other macromolecules to form adducts, hindering DNA replication and transcription, and ultimately, killing the worms^53^ ^-^ ^55^.

Oxamniquine has no killing effect against *S. japonicum* and *S. haematobium* despite them having SULT-OR with 58% (sjSULT-OR) and 71% (shSULT-OR) sequence identity with *S. mansoni* smSULT. In addition, the PAPS contact residues for the three schistosome species are evolutionary conserved^50^ ^,^ ^54^ ^,^ ^55^. Using kinetic analyses to directly compare the enzymatic activities of smSULT-OR, sjSULT-OR, and shSULT-OR with oxamniquine as a substrate, studies have found that smSULT-OR had the highest catalytic efficiency for oxamniquine based on the k_cat_/K_m_ ratio values. When compared to smSULT-OR, sjSULT-OR and shSULT-OR were less active by an order of magnitude and by a factor of one-half, respectively, implying that low levels of catalytic efficiency and diminished turnover of oxamniquine toxic compounds may explain the inefficacy of oxamniquine in killing *S. japonicum* and *S. haematobium* ^54^ ^,^ ^55^. Another study has reported that oxamniquine was unable to productively fit into the sjSULT-OR and shSULT-OR binding pockets, leading to diminished activation of oxamniquine and insufficient production of toxic compounds to kill *S. japonicum* and *S. haematobium* ^50^.

Oxamniquine was previously extensively used as the first-line antischistosomal drug in Brazil, where only *S. mansoni* was prevalent^50^. Hycanthone was also previously used and was effective against the adult worm stage of *S. mansoni* and *S. haematobium*; however it was reported to be carcinogenic^56^ ^-^ ^58^. Resistance against oxamniquine and hycanthone has been observed in the laboratory^51^ as well as in the field^59^ ^-^ ^62^. Mutations in *S. mansoni* smSULT-OR are responsible for oxamniquine resistance in laboratory-derived resistant isolates as well as that observed in the field^55^ ^,^ ^62^ ^-^ ^65^.

Praziquantel is currently the only first-line antischistosomal drug used extensively worldwide for repeated mass chemotherapy in infected individuals, because it is effective against all three schistosome species^66^. This mono-chemotherapeutic strategy for controlling schistosomiasis spread and infection poses challenges as praziquantel is only active against adult schistosomes, ineffective against eggs lodged in the tissues, does not prevent reinfection, and there is a continuous threat of developing praziquantel-resistant parasites^6^ ^-^ ^10^. Evidence for drug resistance due to constant selective pressure through praziquantel mass chemotherapy has been reported both in laboratory populations^6^ ^,^ ^7^ and in the field^8^ ^-^ ^10^.

Sankaranarayanan *et al.* (2021) investigated CRISPR efficiency during the parasitic stages of *S. mansoni* by introducing somatic mutations in the 5.84 kb *SULT-OR* gene (*Smp_089320*) located on chromosome 6 in *S. mansoni* eggs, mother sporocysts (first intramolluscan stage), and adult worms^17^. The aim of this study was to develop an oxamniquine-resistant stable knockout transgenic cell line to test and identify oxamniquine derivatives that have the potential to be effective against all schistosome species, and which can be used in combination with praziquantel to eliminate the emergence of parasite resistance and to enhance overall treatment efficacy. In this study, adult worms had the highest CRISPR efficiency because SULT-OR was highly expressed during the developmental stages, making their chromatin more exposed and accessible to the CRISPR machinery; the worms were followed by sporocysts, while the eggs had the lowest efficiency. Homozygous (biallelic) deletions, induced by CRISPR/Cas9, were predicted to cause resistance to oxamniquine by producing frameshifts, inhibiting *SULT-OR* transcription, or through mRNA degradation via the nonsense-mediated mRNA decay pathway. Additionally, *SULT-OR* knockdown was not observed at the mRNA level, presumably because CRISPR/Cas9 induced mutations in a small fraction of all cells expressing *SULT-OR*. The authors concluded that further optimization of this protocol is required for distinct cell types, including germline cells, in order to derive an oxamniquine-resistant stable knockout transgenic cell line and to identify if variations in protocols would result in varying ranges of mutations and degrees of mRNA knockdown^17^.

### *S. mansoni* α-N-acetylgalactosaminidase (SmNAGAL) gene

In humans, the lysosomal exoglycosidase enzyme, α-N-acetylgalactosaminidase (α-NAGAL), cleaves terminal N-acetylgalactosamine (α-GalNAc) monosaccharides from polysaccharides, glycoproteins, and glycolipids, including those with O-linked carbohydrate sugars attached to serine and threonine residues, and plays an important role in maintaining cellular homeostasis by regulating glycan substrates on carbohydrates, lipids, and proteins^19^ ^,^ ^67^. It belongs to the glycosyl hydrolase family 27 and contains both an α-galactosidase (melibiase)-2 (PF16499) domain and a melibiase-2 C-terminal (PF17450) domain (all enzymatically important catalytic and ligand binding amino acids are conserved within this region)^67^. In humans, deficiency in α-NAGAL activity leads to lysosomal storage disorder known as Schindler disease, which is classified into the following three phenotypic classes: (i) type I disease (a severe infantile neurodegenerative disorder), (ii) type II disease (known as Kanzaki disease and is associated with mild cognitive impairments and angiokeratoma in adults), and (iii) type III disease (associated with a wide myriad of symptoms, including cardiomyopathy, seizures, and autistic disorders)^68^. Fabry disease can also manifest as a consequence of aberrant accumulation of galactose-containing glycolipids^69^.

Using sequence and phylogenetic analyses, Hulme *et al.* (2021) investigated the genome of *S. mansoni* to identify glycosyl hydrolase family 27 orthologs of *Homo sapiens* α-galactosidase and α-NAGAL enzymes^19^. Five glycosyl hydrolase family 27 members were found in *S. mansoni*, but only one, namely SmNAGAL (*Smp_089290*), contained all 13 ligand-binding amino acid residues and both catalytic aspartic acid residues that are crucial for α-galactosidase/α-NAGAL substrate cleavage and activity. Spatial localization of *Smp_089290* revealed its accumulation in neuronal and parenchymal cells as well as in the vitellaria and mature vitellocytes of the adult schistosome^19^.

Using small interfering RNA (siRNA)-mediated knockdown and programmed CRISPR/Cas9 somatic genome editing, Hulme and colleagues further inhibited the newly identified *SmNAGAL* gene (*Smp_089290*) in *S. mansoni* adult worms. *SmNAGAL*-knockdown adult worms displayed inhibited growth and development as well as impaired neuromuscular activity and motility. Female worms displayed diminished egg production, impaired oviposition processes, and produced abnormally shaped eggs^19^. Compared to control worms, siRNA-mediated knockdown of *Smp_089290* significantly inhibited SmNAGAL activity in adult worms, and no significant decrease in α-galactosidase activity was observed. In addition, reductions in the SmNAGAL activity was associated with significant inhibition of adult worm motility and egg production. CRISPR/Cas9-mediated editing of *Smp_089290* in adult worms confirmed the reduction in the egg production. It was concluded that *Smp_089290* acts principally as an α-NAGAL^19^.

### Biomphalaria glabrata allograft inflammatory factor (BgAIF) gene

In humans, allograft inflammatory factor 1 (AIF-1) is an evolutionarily conserved calcium-binding scaffold protein that is predominantly expressed in granular and phagocytic leukocytes^70^ ^,^ ^71^. AIF-1 is an NF-κB inflammation pathway regulator that is important for pro-inflammatory activity and survival of macrophages and plays a role in promoting the activation, proliferation, and migration of B- and T-lymphocytes^71^ ^-^ ^73^.

As an obligatory component of the life cycle of *S. mansoni*, sporocysts adhere to freshwater *B. glabrata* (gastropod snails) to promote larval development^4^. An orthologue of human AIF-1 in *B. glabrata*, namely BgAIF, is highly expressed in *B. glabrata* isolates that are resistant to infection with *S. mansoni* and plays a crucial role in cell-mediated immune responses, hemocyte activation, cellular proliferation, cellular migration, and phagocytosis^74^ ^,^ ^75^. Hemocytes play an important role in cellular and innate immunity in gastropods^4^. Hemocytes found in snails resistant to *S. mansoni* have the ability to encapsulate and destroy schistosome sporocysts^76^ ^-^ ^78^. BgAIF has been hypothesized to activate hemocyte cell adhesion and migration once the schistosome miracidium penetrates the snail tissues^4^ ^,^ ^73^.

Coelho *et al*. (2020) used gene knockout manipulation to target the 2,226 bp-long *BgAIF* gene (accession number BGLB005061) located in the *B. glabrata* embryonic (Bge) cell line, and thereafter transfected *AIF*-knockout Bge cells using *in vitro* transformed *S. mansoni* sporocysts^4^. This Bge cell line exhibited a hemocyte-like phenotype including encapsulation of the larval parasites; however, the cells did not kill the parasites. The *BgAIF*-knockout Bge cells displayed diminished adherence to sporocysts of *S. mansoni,* indicating that the BgAIF protein plays a crucial role in activating Bge cell recognition, migration, and adhesion, and in promoting an early immune response to parasites^4^. CRISPR/Cas9 editing of *BgAIF* gene in germline and somatic tissues of intact *B. glabrata* snails may play a vital role in preventing the transmission of schistosomiasis.

## CHALLENGES

Some of the issues that require attention to improve the overall effectiveness of CRISPR/Cas9 for parasitic worm research include enhancing gene mutation efficiency and optimizing amplicon sequencing techniques to optimally detect both target and off-target genetic variations^79^ ^,^ ^80^. Random large gene deletions in *C. elegans* ^81^ and *Strongyloides stercoralis* ^82^ have been overlooked during amplicon sequencing during CRISPR/Cas9 editing. As a result, it was proposed that undetectable off-target mutations acquired during gene editing could have caused substantial phenotypic alterations observed in both flatworm species at the protein level^79^. Furthermore, both the target and undesired off-target gene mutations were proposed to have collectively influenced the expression of other important genes in downstream pathways, resulting in the observed phenotypic results^79^. This scenario could possibly be linked to the paradoxical findings from a study that showed a significant downregulation of Th2 and Th1 cytokine levels and a reduction in pathophysiological outcomes in hosts that were infected with *S. mansoni ω1*-mutated eggs despite the fact that the eggs had an extremely low *ω1* gene modification frequency (NHEJ (~4.5%) and HDR (0.19%)) (Table 2)^18^; nonetheless, further studies are required to validate this claim.

Another issue to address and overcome is the observed differences in gene mutation efficiency for *S. mansoni ω1* ^18^ ^,^ ^20^, *AChE* ^16^, *SULT-OR* ^17^, and *SmNAGAL* genes^19^. Some of the possible explanations for the observed variations in CRISPR/Cas9 gene editing mutation efficiency are as follows: (i) adult worms have a larger surface area-to-volume ratio than eggs and sporocysts, which may make electroporation more effective for delivering CRISPR components^17^ ^,^ ^79^, (ii) *S. mansoni* eggs have a hardened and tanned outer structure and have serpiginous branching channels, which may obstruct effective transfection^79^ ^,^ ^83^, (iii) compared to eggs and sporocysts, adult worms tend to have an elevated expression level of some of the important NHEJ pathway enzymes, suggesting that DNA repair activities in the NHEJ pathway are more efficient in adult worms^17^ ^,^ ^79^, (iv) the efficacy of CRISPR/Cas9 editing may vary depending on the methodology used for each of the aforementioned genes^79^, and (v) the function or distribution of the targeted gene may influence the pattern of CRISPR/Cas9-induced changes^79^. To facilitate future gene functional studies in schistosomes, more research is needed to improve the efficacy of CRISPR/Cas9 in these parasites during different developmental stages (egg, sporocyst, and adult worm stages).

Some of the disadvantages of using Cas9 nuclease include poor delivery to the target gene site owing to its high molecular weight, off-target effects, undesired immune responses, unexpected repair outcomes, and cellular stress^79^ ^,^ ^80^. Controls that degrade or inhibit Cas9, biomolecule-Cas9 conjugates, and base editors have been developed to overcome these constraints^79^ ^,^ ^80^.

Cas9 is not ideal for parasites containing genomes enriched with AT, which makes the search for a suitable PAM sequence and cloning of repair templates troublesome^80^. Cas9 recognizes 5′-NGG-3′ as the PAM, where “N” can be any nucleotide base, followed by two guanine (G) nucleotide bases, and leaves a blunt double-stranded DNA break^80^. As a possible solution, Cas12a (formerly known as Cpf1) nuclease was proposed to be ideal for parasites with large AT-rich genomes, such as *Plasmodium falciparum* ^80^ ^,^ ^84^. Cas12a is more advantageous than Cas9 because it has a lower molecular weight and requires smaller sgRNA. In addition, Cas12a recognizes thymine (T)-rich 5′-TTTV-3′ as the PAM, where “V” can be any other nucleotide base and leaves a staggered double-strand DNA break^80^. In a recently published study, researchers attempted to mutate the AT-rich genome of the *ω1* gene in *S. mansoni* eggs by using and comparing the efficiency of Cas9 and Cas12a nucleases. The CRISPR/Cas12a group showed the highest knockout efficiency, while the CRISPR/Cas9 group showed the highest transgene insertion/knock-in efficiency (Table 2)^20^.

Cas13 was also proposed as a better alternative to Cas9 and Cas12a because it does not require a specific PAM sequence to cut the RNA, provided that the secondary RNA structure allows for its binding^85^ ^,^ ^86^. Cas13 has the potential to be valuable when performing high-throughput gene knockdowns in parasites that lack the RNAi machinery, such as *T. cruzi*, *Plasmodium* sp., and *Leishmania* sp^80^ ^,^ ^85^ ^,^ ^86^. To date, no study has investigated the efficiency of CRISPR/Cas13 post-transcriptional gene knockout/knockdown in *S. mansoni*.

CRISPR interference/activation (CRISPRi/a) is a non-genome editing tool for transcriptional manipulation and has the potential to be useful in parasitological research. CRISPRi/a uses an enzymatically inactive Cas9, or 'dead' Cas9 (dCas9), to bind to, but not cut, a gene's promoter region^87^. The binding of dCas9 alone can prevent the assembly or progression of the transcription machinery, resulting in gene knockdown^87^. The efficacy of CRISPRi has been demonstrated in *Plasmodium yoelii* ^88^ and *Plasmodium falciparum* ^89^ and findings suggest that dCas9-mediated gene knockdown requires binding to an optimum location that can only be identified through trial and error, and that CRISPRi efficiency might be enhanced by utilizing numerous sgRNAs to tile dCas9 across the promoter region^80^. The question whether CRISPRi/a can be used to inactivate key target genes in *S. mansoni* remains unanswered.

## CONCLUDING REMARKS

CRISPR/Cas9 gene editing technology is currently “the new kid on the block,” which offers an innovative approach to understanding the biological and pathophysiological mechanisms underlying the manifestation of intestinal helminth parasitic infections in humans (Figure 1) (Table 2). It is a valuable tool for scrutinizing the functional role of parasite genes during developmental stages, gender-associated processes, and disease manifestation, and in identifying drug resistant genes to promote the development of immunologically potent drugs and vaccines with high therapeutic efficacy. Based on the ongoing extensive research and clinical validation, the optimized CRISPR/Cas9 gene editing system has the potential to be a powerful tool in the prevention and management of parasitic diseases by not only promoting drug and vaccine development, but also paving the way for the generation of stable genetically engineered parasites and gastropod snails that are resistant to *S. mansoni* sporocysts. 
